# Trophic Garnishes: Cat–Rat Interactions in an Urban Environment

**DOI:** 10.1371/journal.pone.0005794

**Published:** 2009-06-03

**Authors:** Gregory E. Glass, Lynne C. Gardner-Santana, Robert D. Holt, Jessica Chen, Timothy M. Shields, Manojit Roy, Stephen Schachterle, Sabra L. Klein

**Affiliations:** 1 The W. Harry Feinstone Department of Molecular Microbiology & Immunology, The Johns Hopkins Bloomberg School of Public Health, Baltimore, Maryland, United States of America; 2 Department of Botany and Zoology, University of Florida, Gainesville, Florida, United States of America; University of Liverpool, United Kingdom

## Abstract

**Background:**

Community interactions can produce complex dynamics with counterintuitive responses. Synanthropic community members are of increasing practical interest for their effects on biodiversity and public health. Most studies incorporating introduced species have been performed on islands where they may pose a risk to the native fauna. Few have examined their interactions in urban environments where they represent the majority of species. We characterized house cat (*Felis catus*) predation on wild Norway rats (*Rattus norvegicus*), and its population effects in an urban area as a model system. Three aspects of predation likely to influence population dynamics were examined; the stratum of the prey population killed by predators, the intensity of the predation, and the size of the predator population.

**Methodology/Principal Findings:**

Predation pressure was estimated from the sizes of the rat and cat populations, and the characteristics of rats killed in 20 alleys. Short and long term responses of rat population to perturbations were examined by removal trapping. Perturbations removed an average of 56% of the rats/alley but had no negative long-term impact on the size of the rat population (49.6±12.5 rats/alley and 123.8±42.2 rats/alley over two years). The sizes of the cat population during two years (3.5 animals/alley and 2.7 animals/alley) also were unaffected by rat population perturbations. Predation by cats occurred in 9/20 alleys. Predated rats were predominantly juveniles and significantly smaller (144.6 g±17.8 g) than the trapped rats (385.0 g±135.6 g). Cats rarely preyed on the larger, older portion of the rat population.

**Conclusions/Significance:**

The rat population appears resilient to perturbation from even substantial population reduction using targeted removal. In this area there is a relatively low population density of cats and they only occasionally prey on the rat population. This occasional predation primarily removes the juvenile proportion of the rat population. The top predator in this urban ecosystem appears to have little impact on the size of the prey population, and similarly, reduction in rat populations doesn't impact the size of the cat population. However, the selected targeting of small rats may locally influence the size structure of the population which may have consequences for patterns of pathogen transmission.

## Introduction

The impacts of indirect interactions among species in ecosystems have been actively studied for some time e.g. [1]–[3] and the role that predators play in community interactions has received increasing focus in recent years [4]–[6]. A general problem of increasing concern in conservation biology is the decimation of apex predators in ecosystems [7]. The reduction or disappearance of historical levels of predation could have substantial knock-on effects on many ecological processes. This includes the dynamics of infectious disease in which predators play a role in maintaining or improving the health of human populations that can suffer the effects of spillover transmission of both directly transmitted and vector borne pathogens circulating in the prey populations [5], [8], [9].

In large part this interest is justified by observations that predator-prey interactions can produce spatially heterogeneous and counter-intuitive responses among interacting populations. For example, predation by house cats (*Felis catus*) introduced onto islands indicate substantial predation on native ground nesting birds, as well as introduced rodent species (Bonnaud et al 2007), leading to proposals that targeted reductions should be implemented to protect native species. However, Mathias and Catry [10] suggested that the direct impacts of *F. catus* on bird populations may be offset by their predation on other species, such as *Rattus rattus* that also prey on the native avifauna. In experimental manipulations of island populations Raynor and colleagues [11] observed just such an outcome whose effect depended on time and place.

In predator-prey-parasite systems similar arguments have been suggested for interventions. Early analyses concluded that predators generally improved the health of prey (and indirectly human) populations because increased predation shortens the lifetime of infected individuals and shrinks their capacity to spawn further infections [4], [5]. However, more recent studies have identified circumstances where predation increases the prevalence of infection [12]. This occurs when prey have a successful immune response to the infection, there is density-dependent regulation of fecundity and, in the absence of predation, the prey population may be dominated by older individuals who are immune. Predation relaxes density-dependent constraints on fecundity, increasing the supply of new, susceptible hosts.

Urban settings increasing represent as significant portion of the ecosystem experienced by human populations. However, the population interactions of synathropic vertebrate populations remain strikingly under-studied. The depauperate vertebrate communities in human-structured urban environments have many advantages for studying population interactions including predator-prey as well as predator-host-parasite systems. The limited number of species, as well as practical factors, including that many of the species have been adapted to laboratory study makes them tractable for a wide range of studies. Particularly for parasite-associated systems, many of the microorganisms carried by these vertebrates are considered primary candidates for spillover into human populations [8], [13], [14] so these systems are of medical and public health concern.

As an initial step in evaluating urban mammalian population interactions we characterized house cat (*F. catus*) predation on wild Norway rats (*Rattus norvegicus*) in residential neighborhoods in Baltimore, Maryland, USA. Our goal was to determine the impacts of predator and prey interactions on the population characteristics of each species. Studies of synanthropic populations of Norway rats as well as their associated parasites have been conducted in Baltimore for more than 60 years [14]–[25]. Early field experiments involving various perturbations indicated that the quantity and spatial distribution of food resource strongly influenced the abundance of rat populations [17], [18].

House cats (*F. catus*) are the predominant free-ranging mammalian top-predator in this setting. Studies of house cat predatory behavior in Baltimore have supported numerous other reports [10], [26] that suggested cats only occasionally killed rats and rarely have a numerical impact on the prey population, though they can qualitatively affect its structure. Jackson [16] found that Norway rats were food items in only 6.7% of feral cat feces. He also reported that there was no demonstrable relationship between the frequency of cat predation and the abundance of either rats or cats in the alleys. Childs [20], [27] also observed that cat predation on Norway rats was rare — only witnessing five attacks in more than 900 hours of observation. In addition, cats were highly selective in the size of rats they caught — killing rats that were no more than 200 g (86% were 25–100 g), while avoiding larger (up to >600 g) rats.

We sought to confirm these observations and to characterize the impacts of *F. catus* on the primary rodent population in this urban setting. The long-term goal was to determine whether the pattern of predation by house cats might substantially alter the levels of parasite prevalence in their prey populations according to recent theory [12].

## Results

**Figure 1:**
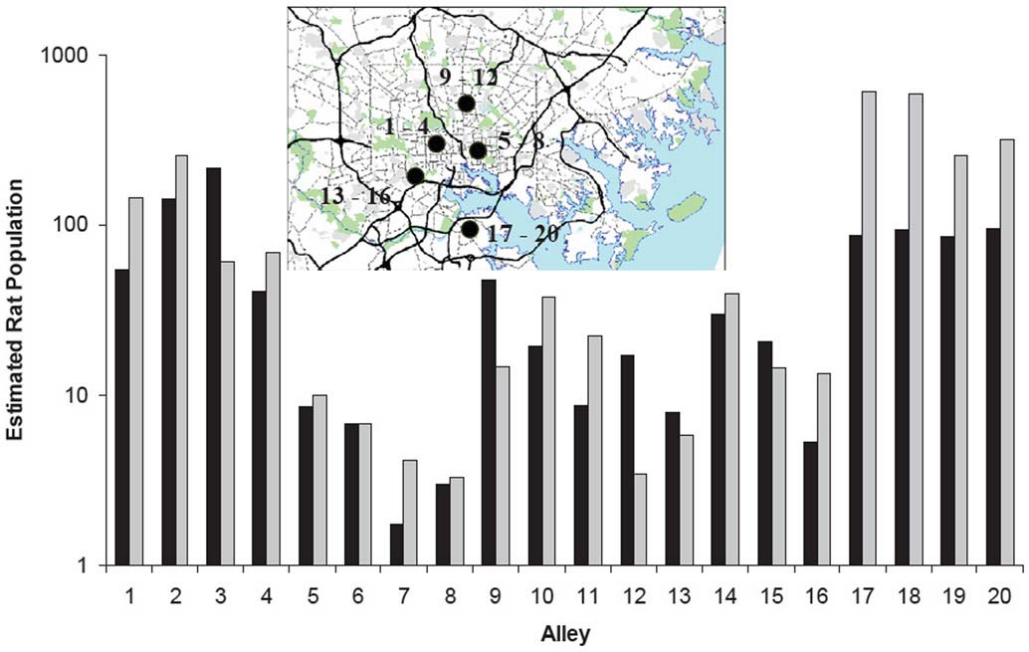
*R. norvegicus* (log scale) trapped in 20 alleys in 2006–2007 (black bars) and 2007–2008 (gray bars). Neighborhoods sampled with corresponding alleys shown in inset.

**Figure 2:**
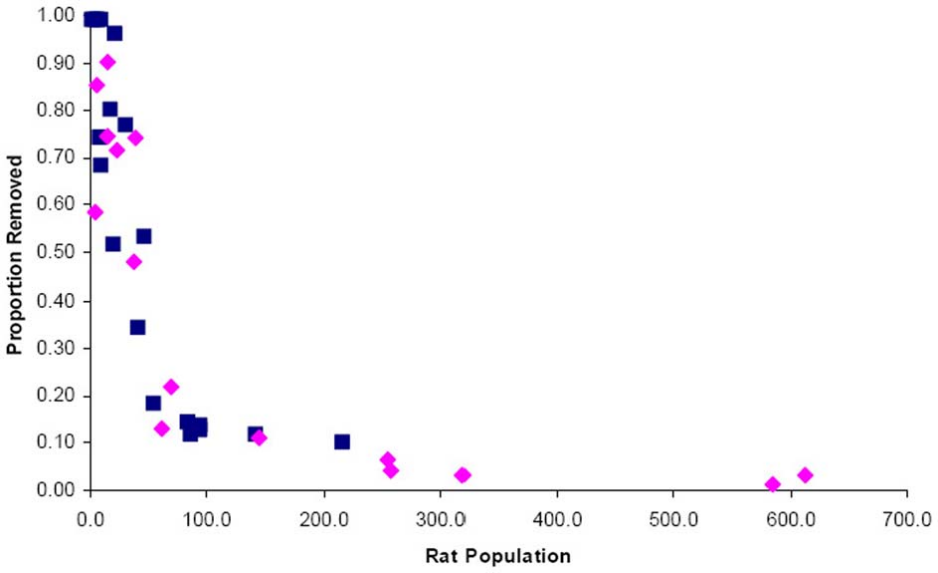
Proportion of rats removed versus estimated *R. norvegicus* population size during year 1 (squares) and year 2 (diamonds).

Sampling and observations were performed during 36 nights from November 2006 through May 2008. A total of 543 rats were removed from 20 alley systems, with 276 removed during the first year and 267 removed during the second. Estimated rat populations ranged from 2 to 584 individuals per alley (Fig. 1). Thus, although the regions selected were considered to be generally infested with rats, there was substantial heterogeneity among even nearby alleys in rat abundance. In none of the alleys was there evidence of spatial clustering for traps catching rats, indicating that the entire area of the alley system was used. This was supported by both direct observation and anecdotal reports from residents. The mean reduction in the estimated rat populations caused by removal trapping in the 20 alleys was 56.1% (range 1.2–92%) during year 1 and 52.0% (range 1.1–96%) during year 2 of sampling (Fig 2). Nearly 2/3 (65%) of alleys had an estimated 20% or more of the trappable rats removed during each year.

**Figure 3:**
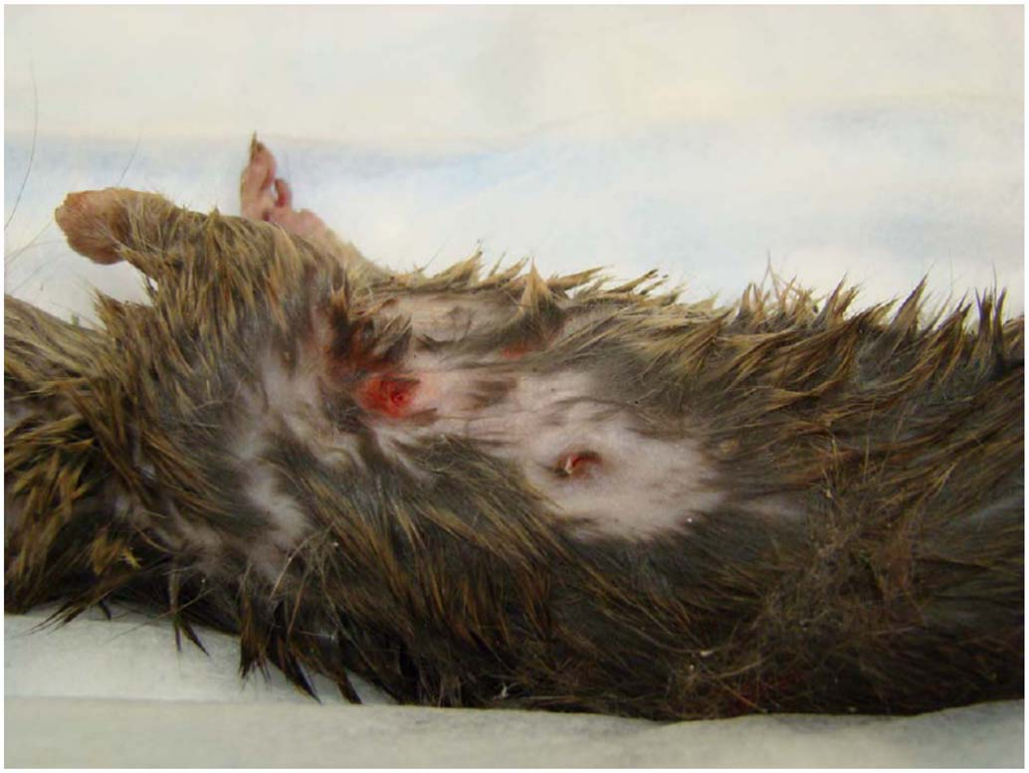
House cat canine puncture to right thoracic cavity of 315 g (body mass) Norway rat.

**Figure 4:**
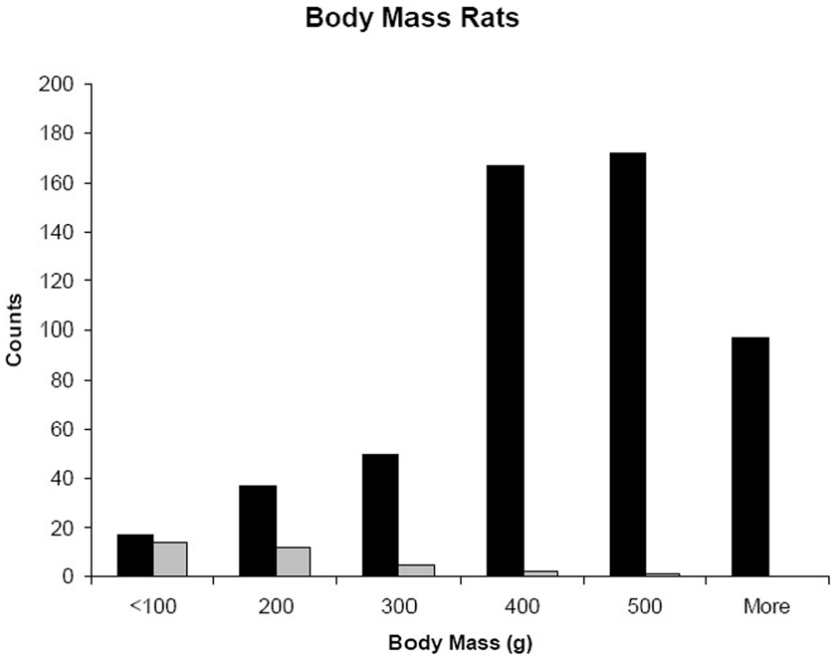
Body sizes of *R. norvegicus* trapped (black bars) and predated (gray bars) by cats in Baltimore.

The average inter-canine distance for 12 housecats was 22 mm and rats with puncture wounds with approximately this spacing were presumed to have been killed by cats (Fig. 3). Cat predation on rats was sporadic and only recorded in 9/20 alleys. A total of 34 predated rats in these alleys were found. Body masses were directly available for 15 rats and were estimated for the remainder. The predated rats were significantly smaller than rats that were trapped in the alleys (estimated body mass = 144.6±17.77 g predated rats vs 388.2±5.32 g trapped rats; p≪0.001; Fig. 4). Cats occasionally selected larger rats (Fig. 3), although more than three-quarters of killed rats were 200 g or less, and 91.1% were smaller than 300 g. The largest rat killed was 508 g.

**Figure 5:**
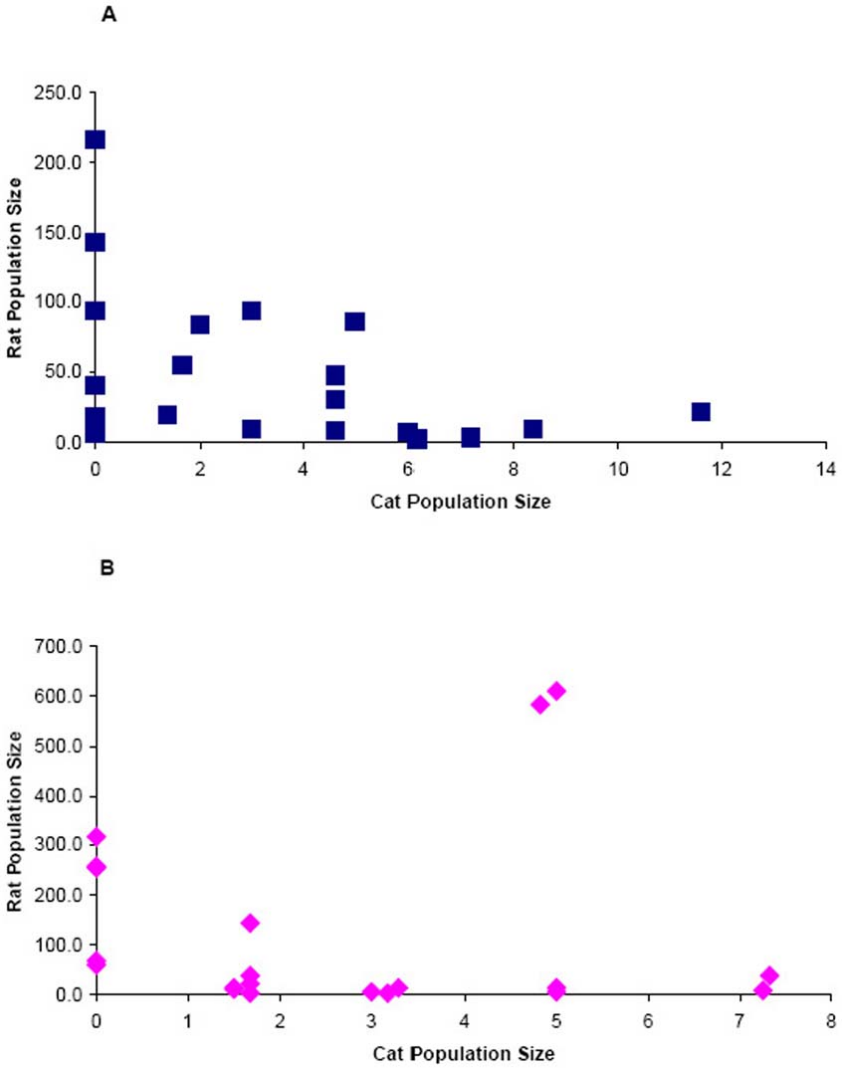
Relationship between rat population size and cat population size during year 1 (A) and year 2 (B).

Estimated cat populations in alleys ranged from 0 to 11.6 individuals (Fig 5A & B). Cat populations appeared unaffected by rat population reductions during both years. The average number of cats prior to rat trapping was 3.5 (±0.74) individuals and was 3.6 (±0.69) cats after rat trapping during the first year. Overall, cat populations tended to be lower during the second year with 2.7 (±0.53) individuals prior to rat trapping and 1.9 (±0.47) animals after trapping. The trend for a decline in the second year might suggest some long term impact of the first year of rat trapping. However, other factors also influenced the cat populations during this time. In at least four alleys under study a local ‘rescue group’ reported that they had removed eight cats in the two weeks prior to our re-sampling rats. Exclusion of these four alleys from the second years' sampling still indicated approximately one fewer cat per alley (−0.9±0.33 cats/alley) during the second year of study.

Although rat populations were estimated to be reduced by more than 50% by removal trapping, their populations appeared unaffected by these perturbations during the second year's followup. Small increases from the first year in estimated rat numbers was observed in 15/20 alleys during the second year (Fig. 1) but the changes were not significant between years (Year effects F (1, 19 df) = 2.89; p<0.25 Randomized block design log(10) transformed numbers; Fig. 1). The average rat population during the first year was 49.6±12.5 rats/alley, while during the second year the estimated populations increased to 123.8±42.2 rats/alley. This increase was driven by a local outbreak of rats in the southern portion of the city where four alleys were sampled (Fig 1). In this area rats increased between years from an average of 89.4 rats/alley to 443.1 rats/alley. At the remaining 16 alleys, the estimated populations were similar to that seen the previous year (44.0±16.8).

There was no relationship between the local abundance of rats and cats in the alleys (Fig 5A & B). The abundance of rats was independent of the numbers of cats found in alleys during both years, as indicated by the absence of significant regression coefficients (all p>0.10) for equations estimating rat populations from cat population sizes.

## Discussion

There is a long history of trying to understand the direct and indirect impacts of predators on prey populations [3], as well as the indirect effects on ecosystem structure [26]. Early models simplified the dynamics of the interactions, ignoring much of the biology that subsequently was found to be important in modifying the dynamics of these systems (e.g. handling time, predator satiation, compensatory mortality). Attempts to understand the more complex indirect dynamics of species interactions demonstrate the rich, and often unpredictable dynamics that arise [11].

The house cat, Norway rat system is of practical interest in that both species commonly are associated with urban areas representing a frequent and widespread portion of a community associated with human populations. In addition, both are reservoirs of pathogens affecting human and other populations [14], [27]–[29]. These species are probably among the best studied of urban vertebrate species both generally, and specifically in the Baltimore region, although detailed knowledge of their ecological interactions in these settings is surprisingly lacking. The patterns observed in the present study are consistent with previous characterizations of the population interactions, extending back more than half a century and suggest this system is remarkably stable, at least on a broad scale.

Estimates of urban rat populations in Baltimore were conducted as early as the 1940's when it was reported that the size of the trappable population in residential areas was approximately 43,000–45,000 [30], [31]. When these surveys were repeated in 2004, the total Norway rat population was essentially unchanged [25]. This global estimate hides some interannual variation and substantial spatial heterogeneity at local levels [22]. However, local populations of Norway rats appear remarkably resilient to most perturbations (Fig 1; [15], [17]). Reduction in the amount of food sources or changes in its spatial distribution appears to be the primary factor that has a rapid and long term impact on the size of rat populations [17], [18].

The apparent extent of predation by cats observed here also is consistent with earlier, local studies [16], [20], and indicate that cats do not rely on rats as their predominant food, but rather scavenge many of the same resources as the rats. As reported in other studies of feral cats [10], [26] they are generalist predators and appear to have relatively little demographic impact on their target populations. In urban areas cats appear even less reliant on rats for food than in more ‘natural’ conditions. This may reflect differences in resource availability between semi-provisioned, urban cat populations [16], [27] and feral cat populations, such as those on islands [10]. It also probably reflects aspects of the prey base. Norway rats reach substantially larger sizes, especially in urban areas [32] than other members of the genus, and are unlikely to be attacked by most house cats [20](Fig 3) compared to smaller congeners such as *Rattus rattus*, whose adult body size, of approximately 200 g, tends to be that of juvenile *R. norvegicus* [22], [32].

The selective nature of cat predation, here, is similar to that reported by Childs [20] with nearly all prey being juvenile (≤200 g) rats, and only occasional predation of larger adult animals. This targeted predation can influence the age structure of the population where recruitment of dispersing young appears influenced by density-dependent social factors [17] and has further relevance downstream in our understanding of pathogen dynamics as many of the rat associated pathogens show an age-dependent pattern of acquisition with much of the transmission occurring near the onset of sexual maturity (ca. 200 g) [14], [19], [21].

Alternative food resources, coupled with feline social behavior [27] may explain the stability of the domestic cat population in the city. Jackson's [16] estimates of cat population size (3.2 cats/alley) in the alleys he surveyed are similar to those reported here more than a half century later. That coupled with observations by Childs [20] indicate that this is a system where the top predator has a relatively little impact on size of the rat population while the cat population does not need to rely on the abundance of rats for its persistence.

The lack of gross demographic impact of predators and prey on each other in this system and the apparent resilience of the rat population to removal perturbation (Fig. 1) may initially suggest that the predator –prey interaction would be of little concern. As such, it has parallels the characterization of micro-organisms that fail regulate their host populations as “trophic garnish” [33]. However, as previously shown [12], [34], these interactions which, at first blush, appear unimportant lead to qualitatively unanticipated outcomes. In this case, by predominantly targeting the weanling and young adult strata of rats (Fig 4) recent theory suggests that cats may induce unanticipated patterns of pathogen prevalence in rat populations. This added complexity of predator behavior and population dynamics injected in host-parasite interactions is not simply an added complication that can be ignored. Rather, it needs to be considered because most vertebrate predators are generalists that are unlikely to be limited by individual prey populations, nor may they limit the overall size of prey populations [5], [35], but nonetheless still alter infectious disease dynamics by altering the relative abundance of different prey classes.

## Materials and Methods

### Ethics Statement

Protocols were approved by the Johns Hopkins Bloomberg School of Public Health Animal Care and Use Committee (RA06H302).

*Rattus norvegicus* were sampled from the central alleys of 20 blocks (five clusters of four adjacent blocks) in high density residential neighborhoods in Baltimore [14], [15], [22], [25] (Fig. 1). These blocks were distributed throughout the city in regions that were reported by the Baltimore City Health Department to have substantial *R. norvegicus* infestations. Protocols for sampling and processing rats have been described previously [22], [25]. Traps were placed along the edges of the alleys adjacent to residential properties and opened at approximate local sunset and were collected the following morning. The address of capture was recorded which located the trap to approximately±3 m. Captured rats were brought to the laboratory and euthanized by CO_2_ inhalation. Standard external body measurements (head and body, tail, hind foot and ear lengths) were recorded to the nearest mm and body mass recorded to the nearest g.

Surveys for predated rats followed previously described methods [20]. Collected predated rats were bagged and brought to the laboratory. Surveys during daytime and evening hours were conducted around denning sites of cats and from actively hunting cats. In addition, carcasses were collected from alleys during surveys of cat populations and rat trapping. Rats that were dead but not directly observed being killed by cats were brought to the laboratory and examined for puncture wounds consistent with cat canine teeth. In the laboratory, standard external measurements were recorded for as much of the rat as was available. Freshly killed rats that showed little tissue loss also were weighed to the nearest g. When major portions of the rats had been consumed, then available standard measurements were used to estimate body mass (±95% CI) using multiple linear regression equations derived from the body measurements of the trapped rats. The body masses of predated rats were compared with the body masses of trapped rats.

Surveys to estimate local cat population sizes were conducted during daylight hours depending on weather conditions. The strategy was to maximize the likelihood of observation. During cold weather, surveys were conducted during the warmest parts of the days to observe cats sunning. During warm weather surveys were conducted either during early morning hours or in late afternoon as cats became active. Two observers walked the length of each alley tallying cats and then repeated the survey while returning. Individual cats could be identified based on pelage patterns and texture [36], as well as sex and body size. Surveys were performed 3–4 days prior to sampling for rats, during rat sampling and then repeated for 3–4 days after rat trapping.

Individual cats were presumed recognizable during single surveys but could be counted multiple times over the course of the entire survey period. Therefore, cat population sizes in each of the alleys were estimated using Noether's method [37]. Estimated cat populations prior to trapping were compared with those during and after trapping to determine the short-term impact of perturbations to the rat population on the estimated size of the cat population. Surveys for cats in each alley was repeated during the second year to characterize the heterogeneity in local populations and to examine the longer term effects of the perturbation of rat removal on cat populations.

Rat populations were estimated from the capture data [38] (pp 20–22). This density estimator was converted to abundance by estimating the area of the central alley in each of the surveyed blocks derived from digital 1∶1000 scale property documents (MdProperty View, Maryland Department of Planning). To determine whether the entire alley was used by rats, the spatial distribution of traps catching rats was compared to the distribution of traps that did not catch rats, using the difference in K-functions [39]. Significant spatial clustering in the difference would indicate that at least a portion of the alleys was not used by rats. The estimated size of the rat population in each alley was used as the denominator to estimate the proportion of the rat population removed during trapping (as a measure of the strength of the perturbation) and population sizes were compared between years to evaluate the long term effects of removal trapping perturbations on the rat population.

Before using parametric statistical methods to test for differences among groups, data were examined for deviations from assumptions for normality. When necessary, transformations were used (e.g. log (10)) to correct for violations in assumptions. Results were back-transformed to original units for reporting.
